# Stress among on-duty firefighters: an ambulatory assessment study

**DOI:** 10.7717/peerj.5967

**Published:** 2018-12-11

**Authors:** Susana Rodrigues, Joana S. Paiva, Duarte Dias, João Paulo S. Cunha

**Affiliations:** 1 Institute for Systems Engineering and Computers—Technology and Science (INESC TEC), Porto, Portugal; 2 Faculty of Engineering, University of Porto, Porto, Portugal; 3 Astronomy and Physics Department, Sciences Faculty, University of Porto, Porto, Portugal

**Keywords:** Occupational health, Firefighters, Stress, Ambulatory assessment, Wearable devices, Work analysis

## Abstract

**Background:**

Stress at work has been broadly acknowledged as a worldwide problem and has been the focus of concern for many researchers. Firefighting, in particular, is frequently reported as a highly stressful occupation. In order to investigate firefighters’ occupational health in terms of stress events, perceptions, symptoms, and physiological reactions under real-world conditions, an ambulatory assessment protocol was developed.

**Methods:**

Seventeen firefighters’ cardiac signal was continuously monitored during an average of three shifts within a working week with medical clinically certified equipment (VitalJacket®), which allows for continuous electrocardiogram (ECG) and actigraphy measurement. Psychological data were collected with a software application running on smartphones, collecting potential stressful events, stress symptoms, and stress appraisal.

**Results:**

A total of 450.56 h of medical-quality ECG were collected, and heart rate variability (HRV) analysis was performed. Findings suggest that although ‘fire’ situations are more common, ‘accidents’ are more stressful. Additionally, firefighters showed high levels of physiological stress (based on AVNN and LF/HF HRV metrics) when compared to normative healthy population values that may not be diagnosed using merely self-reports.

**Discussion:**

The proposed ambulatory study seems to be useful for the monitoring of stress levels and its potential impact on health of first responders. Additionally, it could also be an important tool for the design and implementation of efficient interventions and informed management resolutions in real time. Potential applications of this research include the development of quantified occupational health (qOHealth) devices for real life monitoring of emergency personnel stress reactions.

## Introduction

The existing global challenges of our society, the escalation of crime and violence contribute to the increase of critical incidents; therefore, there is a greater need for security forces and emergency services to intervene (Shaffer, 2010). Firefighting is one of the professions with strong responsibilities towards society safety and well-being, considering that firefighters are the first to respond to a critical incident. Firefighters emergency responsibilities includes not only preventing and combating fire, but also tasks such as supporting in major transport accidents, natural disasters, terrorist attacks, or when special technical help is required (European Agency for Safety and Health at Work, 2011). Society has strong expectations of the ability of firefighting personnel to carry out their responsibilities effectively. However, firefighters perform their typical occupational activities in very hard, unpredictable emergency circumstances, exposing them to severe stress. According to previous findings, working contexts with high levels of stress, are related with increased risks of cardiovascular problems and other work-related illnesses (Vrijkotte, Van Doornen & De Geus, 2000). Furthermore, the effects of stress among firefighters are well recognized (Kales et al., 2007; Perrin et al., 2007).

Stress can be defined as a process, whereas a situation is perceived as exceeding individual’s resources or threatens the person’s well-being (Lazarus & Folkman, 1984). Despite its psychological component, stress also changes the physiological balance of the autonomic nervous system (ANS). The ANS is divided into two main divisions: the sympathetic and the parasympathetic nervous system. Both divisions operate simultaneously and balance each other dynamically in regular situations. During an acute stress event, the sympathetic system is overactive and the parasympathetic system level of activation is decreased. The sympathetic branch increases heartbeat rates, while also increasing the perspiring activity of the adrenal glands and breathing rates, preparing the body to respond, while the parasympathetic system decrease the heartbeat, sweating, and breathing rates (Clifford, Azuaje & McSharry, 2006). This process was firstly called as the ‘fight-or-flight’ response (Cannon, 1935). The chronic exposure to high levels of stress, such as experienced in working situations, can result in a chronic body activation, that can overwhelm the body systems (hormonal, cardiovascular, neural, and muscular systems). This can seriously impact health, for example, by causing long-term damages to the immune system (Taelman et al., 2009).

There are a wide range of physiological indicators (e.g., blood pressure, cortisol, skin conductance), however heart rate variability (HRV) has been foreseen as a reliable indicator of physiological stress reactions (Castaldo et al., 2015). HRV refers to the variations in heart rates or heartbeat intervals, which has been recognized as an instantaneous quantitative measure of the ANS activity related with stress (Zhong et al., 2005).

Developments in stress diagnosis methods are crucial for the understanding of individual functioning during hazardous events and for the design of efficient prevention programs. However, despite the fact that much attention has been dedicated to the study of stress over the years, there are still some limitations with respect to its conceptualization and evaluation. First, traditional stress assessment designs in psychology are frequently retrospective and cross-sectional, only based on self-reported measures (Segerstrom & O’Connor, 2012). As a result, the collected data may be plagued by memory bias and cognitive biases. Second, and as a possible solution to overcome the limitations presented above, laboratory experiments are typically used to assess stress (Zanstra & Johnston, 2011). These controlled conditions avoid retrospective reporting problems and are more rigorous (Smyth & Stone, 2003). However, considering their artificial conditions, results may not represent real-world settings, and their ecological validity is limited. Third, there is a lack of a gold standard stress measurement in either experimental settings or the field (Hovsepian et al., 2015). Finally, studies in this area frequently include clinical populations without contemplating a healthy cohort and/or reference to healthy values (Nunan, Sandercock & Brodie, 2010).

In order to overcome the previous shortcomings in this area and taking into account that stress is a complex issue, interdisciplinary research approaches integrating psychophysiological dimensions of stress that are able to collect data under natural settings are necessary in order to fully investigate stress (Rodrigues, Kaiseler & Queirós, 2015). Ambulatory assessment has been recommended as a novel research method that combines self-reports, and physiological measurements under natural environments (Trull & Ebner-Priemer, 2013). In sum, ambulatory monitoring offers higher ecological validity and higher clinical reliability outcomes than laboratory studies (Houtveen & De Geus, 2009). However, stress research in the field has also been plagued by several problems, including noisy data, the influence of confounding variables, and difficulties in finding discriminative features that can detect and differentiate a stress response from similar physiological responses (Hovsepian et al., 2015).

Based on previous research recommendations, the current study developed a novel multi-method ambulatory stress approach in order to assess firefighters stress events, perceptions and symptoms during their daily work duties. Taking this into account, the current interdisciplinary method consists of a combination of self-reports with electrocardiogram (ECG) data analysis, relying on user-friendly and non-intrusive technology adapted to firefighters’ needs and requirements. According to a recent review on wearable health devices (Dias & Cunha, 2018), wearable ECG devices are of extreme importance for health monitoring because they allow to acquire valuable physiological data without interfering in users normal activities and daily routines. As opposed non-wearable devices have greater dimensions, are static and not comfortable and the awareness of being monitored can strongly interfere with subjects behavior, causing more stress. The major concern when using wearable devices is the associated noise and data quality. The device used in the current study (Vital Jacket®) has the huge advantage of having medical certification, which means that the acquired data has medical quality, although it can be naturally be affected by intensive movement. This is something that has been very difficult to overcome, however the use of these kind of devices contributes for a lower amount of noise artifacts and a higher level of quality of the data. Additionally, the use of softwares that implement machine learning techniques and novel algorithms are also helpful for the removing of movement artifacts (Dias & Cunha, 2018).

To the best of our knowledge, no previous studies were found internationally with this population using a similar approach. Taking this in account, we believe that this study makes a novel and original contribution to the advancement of knowledge in psychophysiological assessment in daily work conditions of firefighters. Findings will particularly increase the understanding of firefighters’ stress experience on duty.

## Methods

**Figure 1:**
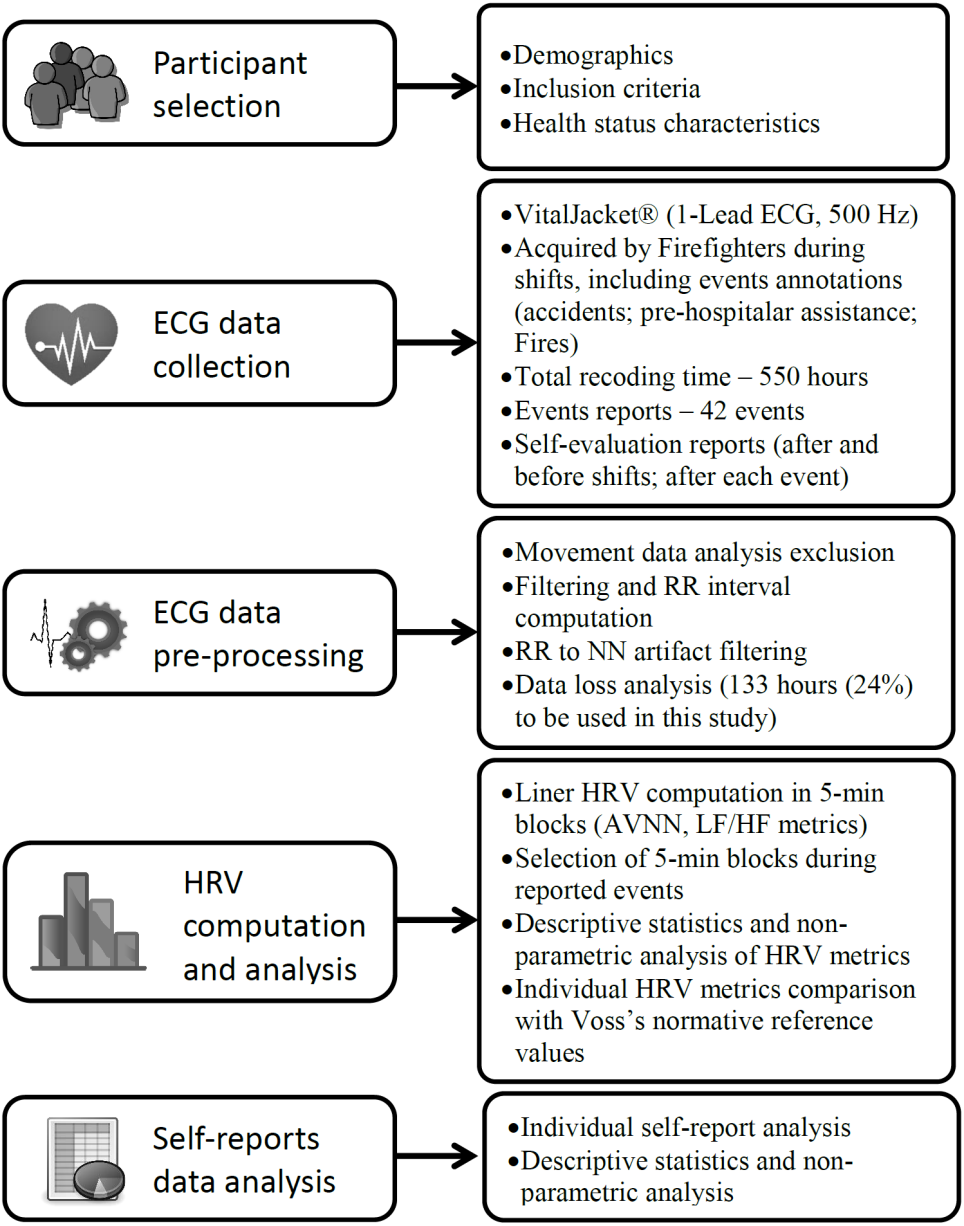
Study method illustration based on Guidelines for Reporting articles in Psychiatry and Heart Rate Variability (Quintana, Alvares & Heathers, 2016).

In order to provide a standardized reporting of methodologies investigating HRV in psychiatry and behavioral sciences, we will present our research method based on the Guidelines for Reporting Articles in Psychiatry and Heart Rate Variability (GRAPH) (Quintana, Alvares & Heathers, 2016). The current method fits the four domains proposed by these guidelines: participant selection, interbeat interval collection, data preparation and HRV calculation. However, small amendments were made to this checklist, considering that our study is multidisciplinary and involves other variables, such as self-report measures, besides HRV metrics. Figure 1 illustrates our method in general.

## Participants

Seventeen firefighters (15 males and 2 females) from a fire department in Portugal voluntarily participated in this study. The age range was 18–54 years (*M* = 29.35 ± 8.85). Regarding the educational background, five had completed primary school, six completed secondary school, and three had a graduation level. The firefighting years of practice ranged between less than one year to 23 years (*M* = 9.41 ± 7.3). Regarding the self-perception of participant’s health status, 47% reported that they have a good health, 35% an excellent health and 18% reported that they have an average health. Regarding physical activity practice, 76% of the sample reported that they practice physical activity regularly. Regarding smoking habits, 53% of the participants smokes.

The exclusion criteria for participating in the study were subjects having a history of cardiovascular disease and/or taking medication that interfere with their cardiovascular function. The study was approved by the University of Porto Ethics Committee (ethical application ref: 29/CEUP/2016). After presenting the study protocol, the participants voluntarily provided written informed consent prior to the study implementation.

## Materials

**Figure 2:**
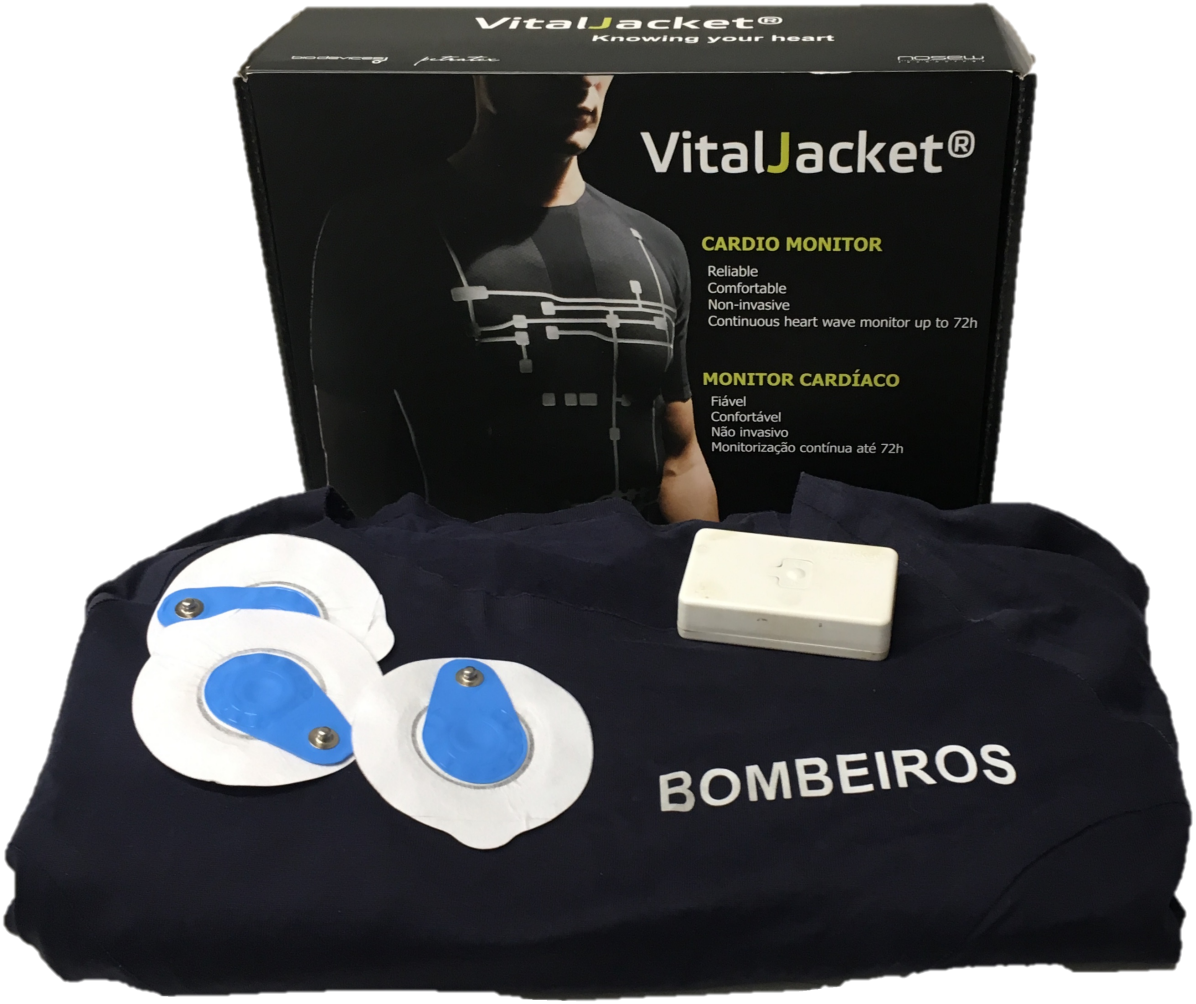
Vital Jacket® equipment.

**Figure 3:**
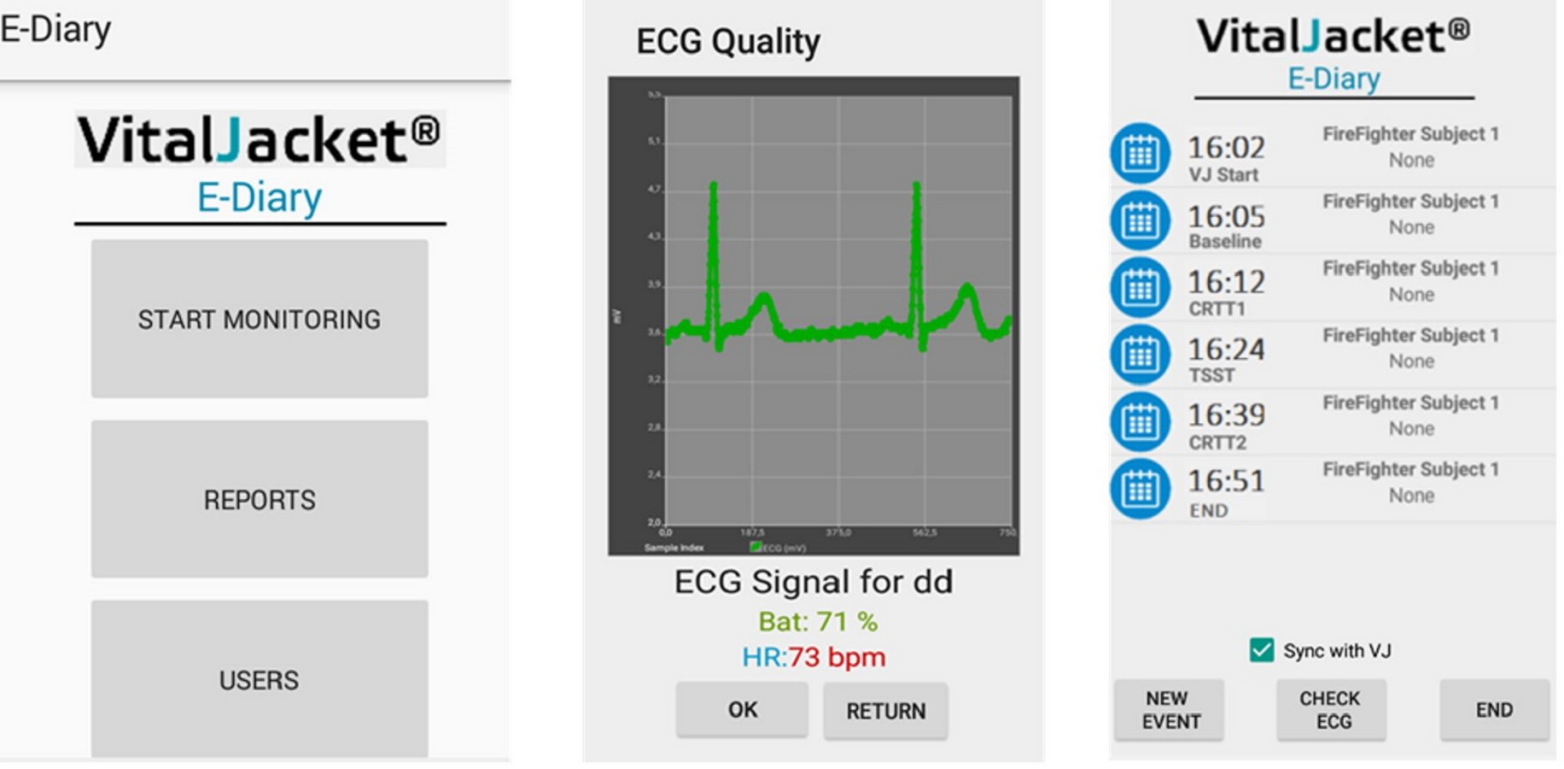
Smartphone application layout.

**Figure 4:**
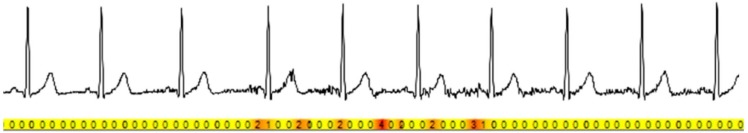
Data acquired from VitalJacket® wearable medical device captured during non-movement and movement. This figure shows an example of the medical grade ECG waveform acquired and below an activity intensity from the accelerometer acquired data. This intensity scale from the accelerometer goes from yellow (no movement, zero value) to red (high movement) showing the level of each participant activity.

A ‘kit’ was specifically developed for this study, including (a) a wearable *t*-shirt that acted like a wearable ECG monitor, VitalJacket® (Cunha et al., 2010; Cunha, 2012) (see Fig. 2), and (b) an electronic diary based on an Android smartphone application (see Fig. 3). The VitalJacket® is a wearable bio-monitoring platform (in the form of a *t*-shirt) able to collect ECG signals in real-time, without affecting daily activities of users. It also contains a three-axis accelerometer system, allowing ECG signal correction for actigraphy profiles, and a Bluetooth transmitter that enables visualisation of the ECG signal in real time and saves all data in a SD memory card. This equipment is certified according to the MDD93/42/EEC medical device directive and holds the European Conformity mark (Biodevices, 2017). Figure 4 shows an example of an ECG trace collected using VitalJacket®.

The electronic diary contained an Android smartphone application that was specifically developed by biomedical engineers for this study. This tool allows to describe events and to rate stress symptoms and perceptions. Additionally, it allows data synchronisation and event marking (Fig. 3). This system pairs with VitalJacket® via Bluetooth and enables the exact annotation of events in the device using ‘radiobuttons’. These events are saved in the device and synchronised with the ECG that is being acquired at that moment. The app stores all the data about the events in an SQL Light DataBase, from where a report of the event data can be generated and exported for processing and analysis, ensuring the synchronisation between those events’ information and the data gathered by the VitalJacket®.

## Measures

### Self-reports

A demographic and a medical questionnaire were used in order to evaluate participants’ current health state. In order to find a measure of chronic stress, the Portuguese version (Mota Cardoso et al., 2002) of the original Perceived Stress Scale (PSS) was implemented (Cohen, Kamarck & Mermelstein, 1983). The validation studies of this scale presented a coefficient alpha reliability above 0.84 (*N* = 332) in Cohen’s and colleagues study and 0.86 (*N* = 200) in Mota Cardoso and colleagues study. In the current study, the Cronbach’s alpha for the PSS was 0.76. The PSS is a 14-item scale ranging from ‘0—never’ to ‘4—very often’. Participants were asked to indicate how often they felt or thought a certain way in the past month. Scores range from 0 to 56, with higher scores indicating more stress.

### Diary information

The Android smartphone application included a stress symptoms questionnaire (Cohen & Williamson, 1988). This instrument included four questions related to physical aspects and four questions related to cognitive aspects. An example of a physical symptom question is ‘I have a stiff neck’; an example of a cognitive symptom question is ‘I lack concentration’. Participants were asked to rate each item on a free scale ranging from ‘0—not felt at all’ to ‘4—extremely felt’. These questions were answered at the beginning and end of the day, aiming to evaluate whether there were alterations in stress symptoms experienced from the beginning to the end of the day. Additionally, a Visual Analogue Scale (VAS) (Lesage, Berjot & Deschamps, 2012) was used after each event and before and after each shift. Participants were asked to rate their perceived stress levels on a 10-level scale, ranging from ‘0—None’ to ‘10—As bad as it could be’. Work stress was classified as high when VAS >6 and low when VAS <3 (Ritvanen et al., 2006). Single–item measures were chosen since they can offer useful information and present numerous benefits (e.g., reduced survey length; respondent burden); for a detailed review of these please see Fisher, Matthews & Gibbons (2016).

The firefighters also provided information regarding the experienced events in an open-ended event question displayed in the smartphone application.

### Ambulatory measures

Following the guidelines presented by the task force of the European Society of Cardiology and the North American Society of Pacing and Electrophysiology (Task Force of the European Society of Cardiology, 1996) a feasible HRV time-domain parameter for stress assessment is the average time between consecutive normal-to-normal (AVNN) heartbeat time intervals. Lower values of this HRV component reflect higher stress (Castaldo et al., 2015). As for spectral domain parameters, both the low-frequency (LF) component (0.04–0.15 Hz) and the high-frequency (HF) component (0.15–0.4 Hz) of all NN intervals provide information about sympathetic nervous system (SNS) and parasympathetic nervous system (PSNS) activity. The LF/HF ratio has been frequently used as an indicator of the overall balance between the sympathetic and parasympathetic systems, respectively. Higher values reflect domination of the sympathetic system, whereas lower values refer to a higher activity from the parasympathetic system (Kaur et al., 2013). HRV values achieved with this population during entire shifts will be compared with normative values obtained by the study conducted by Voss et al. (2015). These authors conducted a study with the largest population of healthy subjects (*N* = 1906) and analyzed age and gender- related HRV differences.

## Procedure

A presentation meeting was organised to present the study. To assess the appropriateness of the study ‘kit’, a pilot study was conducted with two firefighters to validate the methodology’s feasibility and user-friendliness and the accuracy of the data received. Participants underwent ambulatory monitoring on about three days of the same work week (eight hours each shift). At first, all firefighters completed the demographics and medical questionnaire and the PSS. At the beginning of each shift, participants dressed in the VitalJacket®, switched the smartphone on, launched the software application, and filled in the initial stress symptoms questionnaire and the stress VAS. Following this procedure, the firefighters were ready to carry out the equipment for the full working-day periods. After experiencing a stressful situation, the firefighters were required to fill in the event questionnaire on the smartphone, including a description of the event and ratings of stress appraisal, using the stress VAS. At the end of the shift, the firefighters again filled in the stress symptoms questionnaire and the stress VAS.

## Data analysis and Processing

### Event categorisation and correspondent stress perceptions

The event open-ended responses were transcribed verbatim and subjected to an inductive content analysis technique (Maykut & Morehouse, 1994). The data was coded into stressor categories by the first author and then verified by the other authors (Nicholls & Polman, 2007). Then, stressor categories generated for stressor responses were categorised into more general dimensions labelled as ‘fires’, ‘pre-hospitalar assistance’, and ‘accidents’, as recommended in the literature (Pallauf et al., 2011). The frequency and mean values of the stress VAS were calculated considering the intensity and mean intensity of each stressor. This approach is similar to previous research in the area of stress appraisal (Kaiseler, Polman & Nicholls, 2009). Kruskal–Wallis (non-parametric alternative) was used to compare if there were statistical differences across stress VAS scores for the three stress event categories.

### Stress symptoms and self-perceptions

End-of-the-day and beginning-of-the-day stress symptoms mean scores were subtracted in order to achieve an overall mean score, symbolising accumulated stress symptoms over the shift. Internal consistency of the eight questions was calculated using Cronbach’s alphas. A study conducted by Gomes et al. (2012) with a sample of Portuguese firefighters, using the same questionnaire obtained a Cronbach’s alpha for the 8 questions of 0.93. This value provides a coefficient of reliability, and it is used as a measure of internal consistency for participants’ answers. As recommended, these values should be above 0.70 (Pallant, 2013). In the current study, the Cronbach’s alpha for the eight questions was 0.82, with an acceptable reliability (*r* = 0.7).

Paired sample *T*-tests were performed to determine if there were differences between stress symptoms during the end and the beginning of the shift. For stress VAS scales implemented at the end and the beginning of the shift, mean scores were subtracted in order to achieve an overall mean score, representing the evolution of stress perceptions during the shift. Spearman rank order correlations were also used to test if there were relations between the stress symptoms and the perceived stress levels along the shift.

### Physiological stress data

For the extraction of heartbeat information from the ECG recordings, the Biodevices, S.A. ECG analyser (which is the same commercialised by this company to cardiology specialists), was used. This analyser has an algorithm that detects each heartbeat in the ECG recording, detecting the ‘R’ points of the ECG waveform and the RR interval (time between two consecutives ‘R’ peaks in the ECG) was extracted. A simple verification according to the literature was implemented to verify if all the RR intervals were physiologically valid (Clifford, Azuaje & McSharry, 2006). This procedure can eliminate any possible mistake made by the RR interval detection algorithm that can occur in case of a noisy ECG signal. The RR intervals that are physiologically valid are called normal-to-normal (NN) intervals.

Physiological data were collected during the entire shift and during the stressful events. For the event-based approach, AVNN and LF/HF values were computed for each 5-minute ECG segment for each subject, during the period of the event annotation made by the firefighter in the smartphone application. It is important to remind that only ECG valid data was considered after the data quality verification described above. Since the total number of 5-minute ECG blocks among all subjects was not balanced between the three categories (‘accidents’, ‘pre-hospitalar assistance’, and ‘fires’), a number of AVNN and LF/HF 5-minute block values corresponding to the minimum number of 5-minute ECG blocks among the three categories was chosen randomly from the raw samples distribution of AVNN and LF/HF. Hence, a total of 30 blocks of 5-minute ECGs was considered for each condition in the statistical analysis. This allowed to perform statistical comparisons and correlations considering a balanced sample number per condition. Kruskal–Wallis was used to compare the AVNN and LF/HF mean differences for those stress event categories.

For the entire shift analysis, AVNN and LF/HF mean values for each subject were subtracted from the normative values according to the age and gender of each subject. Normative values were obtained from Voss et al. (2015) study with 1,906 healthy subjects.

The statistical analysis was conducted using IBM SPSS AMOS (v.22) software and ECG data analysis was performed using an interface developed in Matlab (R2015a).

## Results

A total of 450.56 h were collected during shifts throughout the fire season in Portugal (July–October 2016). Considering that the combined effect of exercise and mental stress in HRV variables is still not well discriminated by the literature, some adjustments on the analysis had to be made, resulting in a total of 133 h of ECG signal considered suitable for analysis. Hence, in order to remove higher values of movement that may affect HRV changes due to stress, a selection of collected data was made according to a movement threshold measured by the wearable device accelerometer. It is also important to mention that, as stated before, a RR interval validation was made to ensure that the extracted values were physiological accepted. These adjustments enables the reduction of noise artefacts and to exclude wrong values before compute HRV measurement.

The overall sum of PSS results (28 ± 4.99) was in the average (28), considering that the highest possible value is 56, and higher levels suggest more stress. The mean value obtained was 2.0 ± 1.1.

### Event categorisation and correspondent stress perceptions

A total of 42 events were reported by the firefighters. The categorisation of events was divided into three main classes: ‘fires’, ‘pre-hospitalar assistance’, and ‘accidents’ (Table 1). The mean results of the VAS after each event showed that ‘accidents’ were rated as the most stressful events (*M* = 3.16), followed by ‘pre-hospitalar assistance’ (*M* = 2.27) and ‘fires’ (*M* = 1.95). However, results from the Kruskal–Wallis test revealed no significant statistical differences in the stress VAS across the three stress events (*χ*2 = 2.37, *p* = .31).

**Table 1:** Event categories, events, frequency, mean and standard deviation (SD) values of stress Visual Analogue Scales (VAS) after each event.

General dimension/categories	Stress events	Frequency	VAS
			Mean ± SD
Accidents		**6**	**3,16 ± 1,86**
	Road accidents victims	5	
	Accident with an infrastructure	1	
Pre-hospitalar assistance		**15**	**2,27 ± 1,29**
	Glycaemia alterations	12	
	Blood pressure changes	2	
	Patient transportation	1	
Fires		**21**	**1,95 ± 1,05**
	Forest fire	16	
	Transport fire	2	
	Fire in an abandoned house	1	
	Aftermath	2	

**Notes.**

Bold indicates the total frequency of each general dimension and its correspondent mean and standard deviation.

### Stress symptoms and self-perceptions

A total of 34 entries were provided using the smartphone application. Paired sample *T*-test results showed statistically significant differences between the beginning and the end of the shift for all cognitive stress symptoms, with the exception of two physical stress symptoms: ‘stiff neck’ and ‘difficulty keeping the body straight’ (Table 2). There has been an increase of the symptoms from the beginning to the end of the shift, showed by the positive direction of scores between beginning and end of shift (Table 2). Spearman’s rho showed significant positive correlations concerning the difference between Visual Analogue Scales at the end and at the beginning of the shift and the stress symptoms reported, with the exception of ‘unconformable abdominal pain or stomach ache’ and ‘difficulty in controlling reactions’.

**Table 2:** Statistical analysis of differences between end and beginning of the shift for stress symptoms and their correlations with mean difference of VAS along shift (end shift minus beginning of the shift).

Stress symptoms	Mean ± SD^a^	*T*-Test values	Spearman rho
Stiff neck	0.21 ± 0.08	−1.49	0.483^**^
Tiredness in the eyes or heavy head	0.39 ± 0.95	−2.34^*^	0.414^*^
Uncomfortable abdominal pain or stomach ache	0.32 ± 0.73	−2.60^*^	0.339
Difficulty to keep the body straight	0.06 ± 0.89	−0,39	0.639^**^
Lack of concentration	0.29 ± 0.84	−2.05^*^	0.503^**^
Difficulty to think, and make decisions	0.44 ± 0.96	−2.69^*^	0.435^*^
Anxiety	0.15 ± 0.66	−1.30^*^	0.365^*^
Difficulty in controlling reactions	0.21 ± 0.54	−2.23^*^	0.225

**Notes.**

a End shift–beginning of the shift.

* *p* < .05.

** *p* < .005.

SD standard deviation

### Physiological stress data analysis

ECG data were obtained for 17 firefighters. The ECG analysis was performed during the entire shifts and during the main event categories—an event-based approach. Results for the event-based analysis suggested that ‘accidents’ were the most stressful events, considering that AVNN presented the lowest values, followed by the highest values of LF/HF, when compared to the other reported events: ‘fires’ and ‘pre-hospitalar assistance’ (Table 3). These results were in accordance with stress perceptions obtained with the VAS. Kruskal–Wallis revealed statistically significant differences only for LF/HF (*χ*^2^ = 12.54, *p* = .002). In order to test for significant differences in LF/HF between the groups (fires; accidents and pre-hospitalar assistance) Mann–Whitney test was used. Results showed significant differences in LF/HF metric during “fires” (*M* = 2.51 ± 0.98) and “pre-hospitalar assistance” (*M* = 1.92 ± 0.68), *z* = 2.74, *p* = .001, *r* = 0.9. Significant differences were also found for “pre-hospitalar assistance” (*M* = 1.92 ± 0.68), and “accidents” (*M* = 2.85 ± 1.56), *z* =  − 3.29, *p* = .001, *r* = 1.1.

**Table 3:** AVNN and LF/HF mean values and SD divided into the three stress events categorizations (*N* = 30).

	AVNN (milliseconds)	LF/HF
	Mean ± SD	Mean ± SD
Accidents	811.39 ± 145.65	2.85 ± 1.56^*^
Pre-hospitalar assistance	830.24 ± 142.29	1.92 ± 0.68^*^
Fires	823.01 ± 134.05	2.51 ± 0.98^*^

**Notes.**

* Significant differences between conditions “accidents”, “pre-hospital assistance” and “fires”; Kruskal-Wallis, two-tailed; *p* < 0.01.

**Figure 5:**
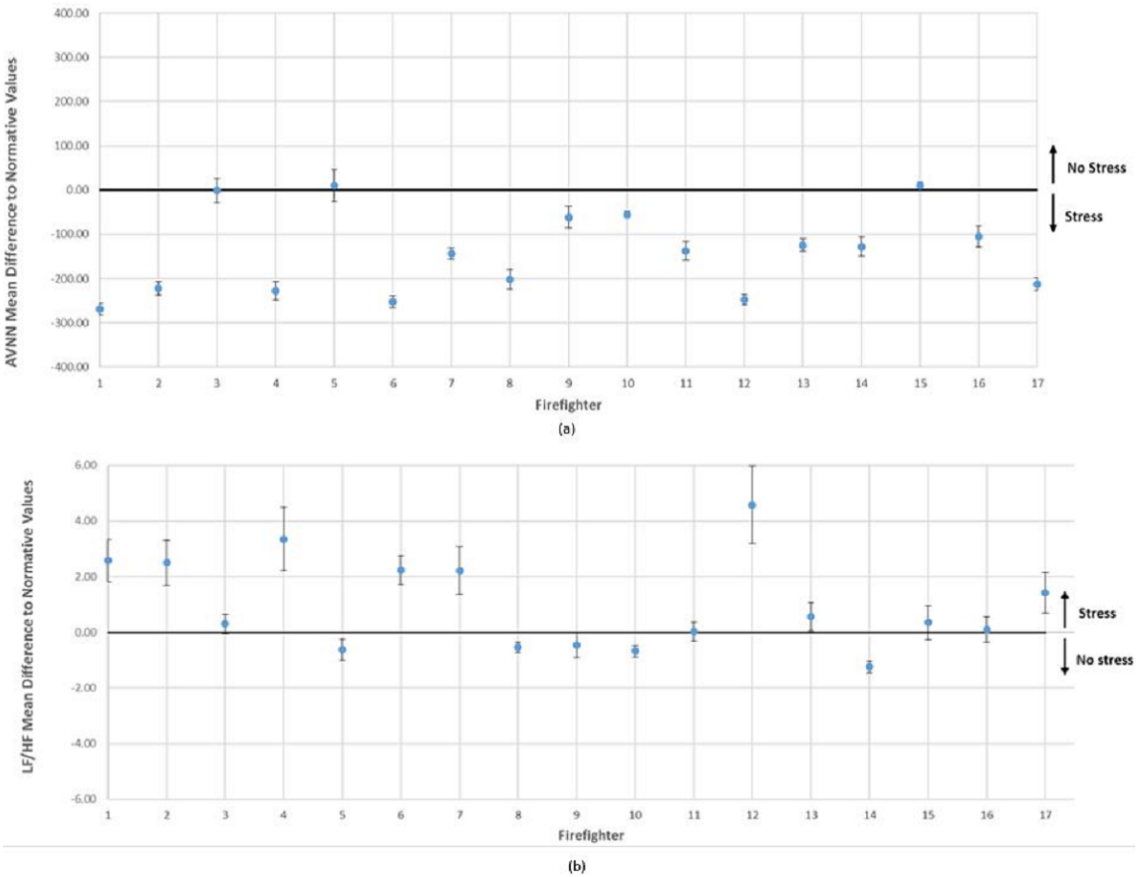
Differences between (A) AVNN and (B) LF/HF obtained during entire shifts and normative values for each subject, individually controlling against baseline HRV for age and gender (Voss et al., 2015).

Results from the ECG analysis conducted during the entire shifts, based on AVNN and LF/HF ratio, are presented in Fig. 5. These results were individually compared with normative values obtained by Voss et al.’s (2015) study with 1,906 healthy subjects, controlling for gender and age (Fig. 4). AVNN results suggested that 82% of participants (14 out of 17 firefighters) presented lower values of this ECG measure, and for LF/HF a total of 71% of the firefighters (12 out of 17) were above the normative values. Overall mean results from the group analysis suggested that AVNN was lower for the firefighters’ sample (*M* = 792.64 ± 92.29) when compared to health subjects’ baseline (*M* = 930 ± 133). Complementarily, LF/HF is higher in firefighters (*M* = 3.82 ± 1.76) when compared to normative values (*M* = 3.33 ± 3.47).

## Discussion

The present study used an ambulatory multi-method approach to assess stress, particularly stress events, perceptions, symptoms, and physiological reactions, considering a combination of self-reports and ECG data. The ambulatory cardiovascular analysis during shifts suggests that firefighters experience high levels of physiological stress when compared to healthy individuals based on the study developed by Voss et al. (2015). There is a lack of studies in the literature concerning normative values of HRV in large healthy populations, not to mention that there is no study indicating these values for a firefighter population.

The reduced values of AVNN during ‘accidents’ and also during shifts propose an increase in cardiac sympatho-excitation, characteristic of stress conditions (Tharion, Parthasarathy & Neelakantan, 2009). This process reflects an increased activation of the cardiovascular system, triggered in order to prepare the body to quickly respond to stress. Therefore, lower values are associated with stress responses (Taelman et al., 2009). On the other hand, increased LF/HF suggests an activation of the SNS as well as withdrawal of PNS activity (Pagani et al., 1997). Therefore, stress is related to an increase in LF/HF ratio (Kaur et al., 2013). Additionally, statistically significant differences were found for LF/HF during the reported events as opposed to AVNN, suggesting that this measure could be more sensitive for the identification of stress, considering that this parameter is directly related to sympathetic activation (Traina et al., 2011). Complementarily, results obtained from PSS, used as a measure of chronic stress suggest a moderate level of this measure, potentially indicating the presence of a chronic stress state (Cohen, Kamarck & Mermelstein, 1983).

When examining the differences between event self-perceptions, it is interesting to note that ‘accidents’ were reported as the most stressful (*M* = 2.85 ± 1.86). However, the differences were not statistically relevant, probably due to the reduced sample used. These results were also found in a similar study with firefighters (Pallauf et al., 2011). This suggests that, although ‘fire’ situations are more common, they are not the most stressful. One possible explanation for ‘accidents’ being the most stressful events could be that firefighters are usually first on the scene of accidents, which are unpredictable situations that usually involve people’s lives. However, it is important to consider that stress self-perceptions, based on the VAS, presented very low values, considering that the scale ranged from 1 to 10 and the overall mean of this measure was 2.38. The possible mismatch between self-report levels after events and their physiological responses could be explained by the fact that the reported events (mainly ‘fire’ situations) are commonly experienced by firefighters, particularly in the course of the fire season, when the data were collected.

Complementarily to stress perceptions and physiology, stress symptoms were also analysed. The stress symptoms questionnaire showed an increase of stress symptomatology from the beginning of the shift to the end, with almost all stress symptoms positively correlated with perceived stress level differences based on VAS means. In fact, VAS stress levels reported by participants also increased from the beginning to the end of shifts. These results are in line with previous findings obtained with this population, showing the cumulative effects of stress throughout the day (Gomes et al., 2012). Additionally, these effects are more evident for cognitive stress symptoms, considering that all of them significantly increased from the beginning of the shift to the end. Accordingly, a study conducted with 21 firefighters concluded that stress has an impact on cognitive functioning, which could explain the maladaptive responses observed during real fires (Robinson et al., 2013). These should be of great concern for firefighters’ administrators and clinicians, providing important insights for the design of tailored stress-management programs.

The current study has some methodological limitations particularly: the reduced sample size that limits the generalizability of results and short time duration of the data collection procedures (only an average of three shifts were monitored). Hence, further research is required, using larger samples and different professionals for longer periods (more shifts and including days off) and comprising longitudinal designs. Regarding the wearable device used for ECG monitoring there were also some technological limitations. Firstly, the fact that the wearable *t*-shirts need to be washed after used, due to the sweat and transpiration. Secondly, there were two cases where the gel-electrode was displaced due to the transpiration, leading to artifacts in the ECG data. Considering these limitations, our research group has been working on the development of a new device version with a reduced skin patch form-factor (Cunha, 2016). Despite the current limitations, we do believe in the potential of this study, considering that the information we have collected from the firefighters health status could be helpful to prevent limit situations during real duties, caused by extreme stress (overexaustion; heat stress). This information could be sent in real time to fire commanders and this could help on the management of teams in the field.

## Conclusions

In sum, there is some evidence that stress is part of firefighters’ routines, but they may not be truly aware of their stress levels; therefore, they are unlikely to ask for help. Stress in the workplace could be problematic if not managed well (Schultz & Schultz, 2010). Therefore, further attention should be dedicated to firefighters’ occupational health by collecting not only information about what causes stress but also concerning its real impact on psychological and physical health. This study reinforces the importance of firefighter research for the monitoring of stress levels and its potential impact on health and the design of intervention plans and programmes adapted to this population’s real needs. Moreover, the multi-dimensional and complex nature of stress for first responders requires that administrators, and associated policy makers take a corresponding and comprehensive method for the management of stress in these hazardous occupations (Reynolds & Wagner, 2007). Such a comprehensive approach to stress management will improve the well-being, safety, and productivity not only of the individual workers but also the communities they serve.

Findings from the current study provide a novel contribute in the area of occupational health, particularly for a better understanding of stress processes. A novel and accurate methodology was developed and its feasibility was showed among hazardous professionals working under real-world environments.

##  Supplemental Information

10.7717/peerj.5967/supp-1 Supplemental Information 1 Firefighters raw data and metadata

## List of Abbreviations

ANS: Autonomic Nervous System
AVNN: Average of Normal-to-Normal Intervals
ECG: Electrocardiogram
HF: High Frequency component
HRV: Heart Rate Variability
LF: Low Frequency component
LF/HF: Ratio of Low Frequency and High Frequency power band
PSNS: Parasympathetic Nervous System
PSS: Perceived Stress Scale
qOHealth: Quantified Occupational Health
SNS: Sympathetic Nervous System
VAS: Visual Analogue Scale

## Additional Information and Declarations

### Field Study Permissions

The following information was supplied relating to field study approvals (i.e., approving body and any reference numbers): Field experiments were approved by the University of Porto Ethical Comission and the Chief Command of the Portuguese firefighter department.

### Data Availability

The following information was supplied regarding data availability: The raw data are included in the Supplemental Files.
